# The Science and Art of Obstetrics

**Published:** 1890-12

**Authors:** 


THE SCIENCE AND ART OF OBSTETRICS. By THEOPHILUS PARVIN, M. D., L. L. D., Professor of Obstetrics and Diseases of Women and Children in Jefferson Medical College, Philadelphia, and one of the Obstetricians to the Philadelphia Hospital. Second edition, revised and enlarged. Illustrated with 231 wood-cuts and a colored plate; pp. xvi.—704. Philadelphia: Lea Brothers & Co. 1890. Price, cloth, $4.25; leather, $5.25. Buffalo: Peter Paul & Bro.

The second edition of this work, following so soon after its first publication, demonstrates its real value and the favor bestowed upon it by the profession. We regard it as the most valuable text-book for the student of medicine yet published. The author has been a most successful teacher for a long period, and the discipline and training of the lecture-room are observed in the clearness with which the often obscure principles of the science and art of obstetrics is presented to the comprehension of the under-graduate. We think this feature is one of the strongest in the work, and commend it especially to teachers.

When the first edition was issued, we favorably reviewed it, and commended it to the profession. The present edition is greatly improved, and embodies all the advances made in this important department of medicine up to the time of its publication. These changes have been accomplished without material increase in bulk, while they have been used with sufficient thoroughness to entitle the volume to be regarded as a new work.

The author, in the section on the curative treatment of eclampsia, says: "Bleeding was formerly regarded by most authorities as the essential treatment, but in recent years it is absolutely rejected by some of the best obstetric teachers." "Those who always bled in eclampsia were certainly wrong, but is it quite sure that those who never bleed were invariably right?" We think if the results of treatment, in which morphia, chloral hydrat. and other drugs were used, were compared with others of the old régime, it would be found that recoveries are not any more frequent, and we doubt if as frequent as formerly. The profession has departed too far from venesection, and must, in a measure at least, return to it.

The chapters on the mechanism of labor are full and clear, and the author's views are in accordance with the latest opinions on these important subjects.

The section on antisepsis is too brief to fully compass a subject of such vast importance. The attention directed to personal cleanliness and the use of germicides, marks an important era in obstetrical practice. The author favors the free use of antiseptics before, during, subsequent to labor, and, to a degree, in the specific instructions given in the pathology of the puerperal state, makes amends for the brief reference given to antisepsis in the chapters above referred to.

The entire work is from the pen of a thorough student and an accomplished obstetrician, and will receive from the profession the endorsement it so richly deserves.

